# High-dose intravenous iron reduces myocardial infarction in patients on haemodialysis

**DOI:** 10.1093/cvr/cvab317

**Published:** 2021-12-07

**Authors:** Mark C Petrie, Pardeep S Jhund, Eugene Connolly, Patrick B Mark, Michael R MacDonald, Michele Robertson, Stefan D Anker, Sunil Bhandari, Kenneth Farrington, Philip A Kalra, David C Wheeler, Charles R V Tomson, Ian Ford, John J V McMurray, Iain C Macdougall, for the PIVOTAL Investigators and Committees

**Affiliations:** BHF Cardiovascular Research Centre, University of Glasgow, 126 University Place, Glasgow G12 8TA, UK; BHF Cardiovascular Research Centre, University of Glasgow, 126 University Place, Glasgow G12 8TA, UK; BHF Cardiovascular Research Centre, University of Glasgow, 126 University Place, Glasgow G12 8TA, UK; BHF Cardiovascular Research Centre, University of Glasgow, 126 University Place, Glasgow G12 8TA, UK; Changi General Hospital, Singapore, Singapore; Robertson Centre for Biostatistics, University of Glasgow, Glasgow, UK; Charite, Universitatsmedizin Berlin, Berlin, Germany; Hull and East Yorkshire Hospitals NHS Trust and Hull York, Medical School, Hull, UK; Lister Hospital, Stevenage, UK; Salford Royal NHS Foundation Trust, Salford, UK; University College London, London, UK; George Institute for Global Health, Sydney, Australia; Freeman Hospital, Newcastle upon Tyne, UK; Robertson Centre for Biostatistics, University of Glasgow, Glasgow, UK; BHF Cardiovascular Research Centre, University of Glasgow, 126 University Place, Glasgow G12 8TA, UK; Department of Renal Medicine, King’s College Hospital, London, UK

**Keywords:** Myocardial infarction, Haemodialysis, Iron, PIVOTAL

## Abstract

**Aims:**

To investigate the effect of high-dose iron vs. low-dose intravenous (IV) iron on myocardial infarction (MI) in patients on maintenance haemodialysis.

**Methods and results:**

This was a pre-specified analysis of secondary endpoints of the Proactive IV Iron Therapy in Hemodialysis Patients trial (PIVOTAL) randomized, controlled clinical trial. Adults who had started haemodialysis within the previous year, who had a ferritin concentration <400 μg per litre and a transferrin saturation <30% were randomized to high-dose or low-dose IV iron. The main outcome measure for this analysis was fatal or non-fatal MI. Over a median of 2.1 years of follow-up, 8.4% experienced a MI. Rates of type 1 MIs (3.2/100 patient-years) were 2.5 times higher than type 2 MIs (1.3/100 patient-years). Non-ST-elevation MIs (3.3/100 patient-years) were 6 times more common than ST-elevation MIs (0.5/100 patient-years). Mortality was high after non-fatal MI (1- and 2-year mortality of 40% and 60%, respectively). In time-to-first event analyses, proactive high-dose IV iron reduced the composite endpoint of non-fatal and fatal MI [hazard ratio (HR) 0.69, 95% confidence interval (CI) 0.52–0.93, *P* = 0.01] and non-fatal MI (HR 0.69, 95% CI 0.51–0.93; *P* = 0.01) when compared with reactive low-dose IV iron. There was less effect of high-dose IV iron on recurrent MI events than on the time-to-first event analysis.

**Conclusion:**

In total, 8.4% of patients on maintenance haemodialysis had an MI over 2 years. High-dose compared to low-dose IV iron reduced MI in patients receiving haemodialysis.

**EudraCT Registration Number:**

2013-002267-25.

## Graphical Abstract

**Graphical Abstract:**
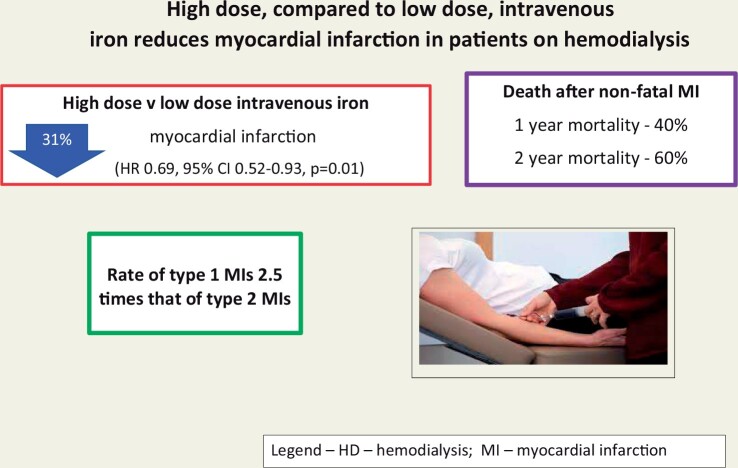

## 1. Introduction

Contemporary data on the incidence and outcomes of myocardial infarction (MI) in patients on maintenance haemodialysis are sparse. MIs in patients receiving dialysis for end-stage kidney disease (ESKD) can be type 1 MIs (classical plaque rupture with thrombus formation) or type 2 MIs (secondary to ‘supply-demand mismatch’ with or without underlying coronary disease).

Concerns have been expressed that intravenous (IV) iron could increase the prevalence or severity of coronary artery disease, and even increase coronary artery events. These concerns have emerged from observational studies in humans, animal studies, and small mechanistic studies.^1–4^ Others have suggested that IV iron may not increase coronary events^5^ but there are no data to suggest that IV iron might reduce MIs. In the Proactive IV Iron Therapy in Hemodialysis Patients trial (PIVOTAL), we compared a regimen of IV iron administered proactively in a high-dose regimen, with a low-dose regimen, administered reactively.^6^ MI was an adjudicated outcome [type 1, type 2, ST-elevation MI (STEMI), non-ST-elevation MI (NSTEMI)] and was a pre-specified secondary endpoint in the trial. Here, we describe the rates and prognostic significance of MI in patients on maintenance haemodialysis and the effect of the high- vs. low-dose IV iron therapy regimens on MIs.

## 2. Methods

The design, baseline characteristics, and main results of PIVOTAL have been published.^6,7^ In summary, 2141 adults who had started haemodialysis within the previous year, who had a ferritin concentration <400 μg per litre and a transferrin saturation <30%, and who were receiving an erythropoiesis-stimulating agent were enrolled. Patients were randomized in a 1:1 ratio, to receive open-label high-dose IV iron administered proactively or low-dose IV iron administered reactively. Ferritin concentration and transferrin saturation were measured monthly and the results were used to determine the monthly dose of iron sucrose. In the high-dose group, 400 mg of iron sucrose was prescribed, with safety cut-off limits (ferritin >700 μg per litre or transferrin saturation >40%) above which further iron was withheld until the next blood test 1 month later. Patients in the low-dose group received 0–400 mg of iron sucrose monthly to maintain ferritin ≥200 μg per litre and transferrin saturation ≥20%, in line with current guidelines. The protocol required the use of an erythropoiesis-stimulating agent in a dose sufficient to maintain a haemoglobin of 100–120 g per litre, but otherwise, patients were treated according to usual practice. The trial was reviewed and approved by the ethics committee. Each patient provided informed consent. The trial conformed to the principles outlined in the Declaration of Helsinki.

### 2.1 Baseline information related to MI

Investigators were asked about the presence of prior MI and other cardiovascular comorbidities on an electronic case report form. The use of cardiovascular medications, including statins, renin–angiotensin system blockers, and beta-blockers, were documented.

### 2.2 Clinical outcomes

The primary outcome of the trial was the composite of MI, stroke, hospitalization for heart failure, or death from any cause, analysed as time-to-first event. MI was a pre-specified secondary outcome. For this article, the outcomes of *time-to-first* MI (type 1 or type 2 MI, STEMI, or NSTEMI) and the composite outcome of MI and death due to MI were reported. In addition to *time-to-first* MI, we also performed a *post hoc* analysis of total (first and *recurrent*) MI events, to account for the cumulative burden of events over time. We also performed a *post hoc* analysis of mortality after (initially) non-fatal MI.

### 2.3 Adjudication of outcomes

All potential endpoints and all deaths were adjudicated by an independent committee, blinded to treatment allocation. The endpoint charter is included in the main results paper.^6^ The endpoint charter is based on the Food and Drug Administration (FDA)-endorsed Standardized Data Collection for Cardiovascular Trials Initiative (SCTI).^8^ For the confirmation of MI, there was a requirement for a rise and/or fall of cardiac biomarkers (preferably cardiac troponin) with at least one value above the 99th percentile upper reference limit and with at least one of the following: symptoms of myocardial ischaemia; new, or presumed new, significant ST-segment T-wave (ST-T) changes, or new left bundle branch block; development of pathological Q waves on the electrocardiogram; imaging evidence of new loss of viable myocardium or new regional wall motion abnormality; and identification of an intracoronary thrombus by angiography or autopsy. Two independent reviewers adjudicated each potential event with discordant results being discussed at a consensus committee meeting involving all adjudicators (comprising cardiologists, a renal physician, and a stroke physician).

### 2.4 Statistical analysis

The time-to-event analyses of the primary, secondary, and *post hoc* outcomes were performed in the intention-to-treat population using Cox proportional hazards regression, with treatment group and stratification factors [(dialysis catheter vs. arteriovenous fistula or graft), diagnosis of diabetes (yes vs. no), and duration of haemodialysis treatment (<5 months vs. ≥5 months)] as explanatory variables. The Kaplan–Meier method was used to estimate mortality rates and cumulative incidence functions corrected for the competing risk of deaths not included in the outcome of interest. Recurrent events were analysed using the proportional means model of Lin *et al*. and described in the form of mean frequency functions (method of Ghosh and Lin^9^). Baseline characteristics were summarized as means and standard deviations, medians and lower and upper quartiles, or counts and percentages as appropriate. *P*-values for between-group differences based on two-sample *t*-tests or χ^2^ tests/Fisher’s exact tests, as appropriate, are provided. Analyses were performed using SAS software, version 9.4 (SAS Institute) and R version 3.6.0.

## 3. Results

A total of 2141 eligible men and women were randomized. In total, 180 (8.4%) patients experienced at least one confirmed fatal or non-fatal MI during the median follow-up of 2.1 years (maximum 4.4 years).

### 3.1 Patients experiencing a MI vs. those not experiencing a MI: baseline characteristics

Patients who had a fatal or non-fatal MI were older (67 vs. 62 years of age, *P* < 0.001) and more often Asian than other races (*Table 1*). Patients who had a fatal or non-fatal MI were more likely to have a history of previous MI, previous heart failure, diabetes, and peripheral artery disease than those who did not experience an MI. Patients with fatal or non-fatal MIs were more likely to have a diabetic or renovascular cause of renal failure than those who did not have an MI. Patients with a type 2 MI had higher systolic blood pressures than those with a type 1 MI (156 mmHg vs. 145 mmHg, *P* = 0.01, Supplementary material online, *eTable S1*).

**Table 1:** Patients with a fatal or non-fatal MI vs. those not experiencing an MI: baseline characteristics

	MI (*N* = 180)	No MI (*N* = 1961)	*P*-value
Age (years)	67.0 (12.3)	62.4 (15.2)	<0.001
Male sex (%)	70	64.9	0.17
Race (%)			0.03
White/European	81	79	
Black/African descent	5	9	
Asian	13	8	
Other	2	3	
BMI (kg/m^2^)	28.8 (5.8)	28.7 (7.0)	0.92
Systolic BP (mmHg)	147 (25)	144 (24)	0.09
Median duration of dialysis (months)	4.9 (2.6–7.9)	4.8 (2.8–8.3)	0.8
History (%)			
Hypertension	81	72	0.05
Atrial fibrillation	11	7	0.03
MI	21	7	<0.001
PVD	16	8	0.002
Heart failure	10	3	<0.001
Stroke	12	8	0.12
Diabetes	65	42	<0.001
Aetiology of renal failure			<0.001
Hypertension	7	11	
Diabetic nephropathy	51	32	
Glomerular disease	11	19	
Tubulointerstitial disease	7	10	
Renovascular disease	15	6	
Polycystic kidney disease	1	6	
Unknown	5	10	
Smoking status (%)			
Never	54	64	0.04
Previous	30	25	
Current	16	11	
Laboratory measurements			
Haemoglobin	105 (13)	106 (14)	0.73
Ferritin	208 (138–294)	217 (133–304)	0.45
Transferrin saturation	20 (15–24)	20 (16–24)	0.45
C-reactive protein	8 (4–17)	6 (3–14)	0.04
Cardiovascular medications (%)			
β-Blocker	48	44	0.25
ACE inhibitor	12	18	0.07
ARB	8	12	0.16
Any diuretic	47	43	0.34
Statin	75	58	<0.001
Any antiplatelet agent	68	43	<0.001

### 3.2 Rates of MIs

#### 3.2.1 Time-to-first event analysis

Rates of fatal and non-fatal type 1 MIs (*n* = 142, 3.3/100 patient-years) were 2.5 times greater than fatal and non-fatal type 2 MIs (*n* = 57, 1.3/100 patient-years) (*Table 2*). Fatal and non-fatal NSTEMIs (*n* = 141, 3.3/100 patient-years) were more than 6 times more common than fatal and non-fatal STEMIs (*n* = 22, 0.5/100 patient-years).

**Table 2:** Event rates—time-to-first event and recurrent (first and subsequent) events analyses

	*n* (% of total in patients in trial)	Rate/100 patient-years
Time-to-first event analyses		
Fatal or non-fatal MI	180	4.2
Fatal or non-fatal type 1 MI	142	3.3
Fatal or non-fatal type 2 MI	57	1.3
Fatal or non-fatal STEMI	22	0.5
Fatal or non-fatal NSTEMI	141	3.3
Recurrent (first and subsequent) event analyses		
Fatal or non-fatal MI	259	6.1
Fatal or non-fatal type 1 MI	193	4.5
Fatal or non-fatal type 2 MI	65	1.5
Fatal or non-fatal STEMI	24	0.6
Fatal or non-fatal NSTEMI	200	4.7

#### 3.2.2 Recurrent event analysis

Of the total number of MIs (*n* = 259), 79 (30.5%) were subsequent (i.e. not first events). Most of the recurrent MIs were type 1 MIs (*n* = 193) with few type 2 MIs (*n* = 65).

### 3.3 Death due to MI

MI was the cause of death in 5% of patients who died of any cause (Supplementary material online, *eTable S3*). Fourteen percent of cardiovascular deaths were due to MI.

### 3.4 Mortality after non-fatal MI

Mortality after a non-fatal MI was 40% at 1 year and 60% at 2 years (Supplementary material online, *eFigure S1*). Mortality was similar for type 1 MIs and NSTEMIs but appeared to be higher for STEMIs (although numbers of these were small) (Supplementary material online, *eFigure S1*).

### 3.5 Effect of high-dose iron vs. low-dose iron on fatal and non-fatal MIs

**Figure 1:**
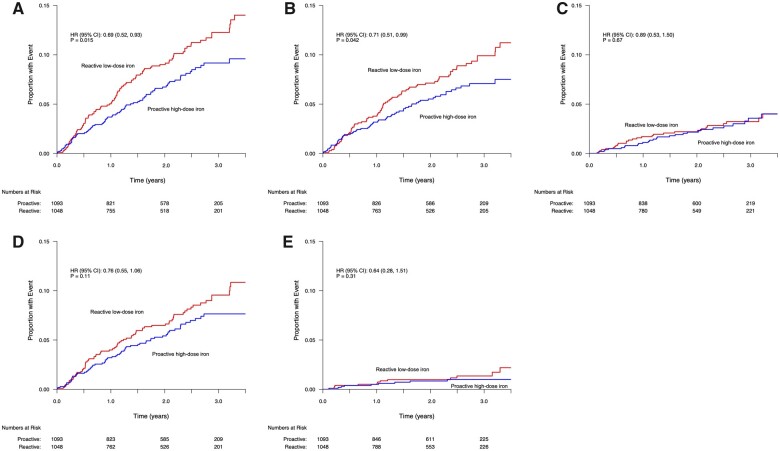
Effect of high- vs. low-dose IV iron on fatal and non-fatal MI (time-to-first event analysis). (*A*) Fatal and non-fatal MI; (*B*) type 1 MI; (*C*) type 2 MI; (*D*) NSTEMI; and (*E*) STEMI.

**Figure 2:**
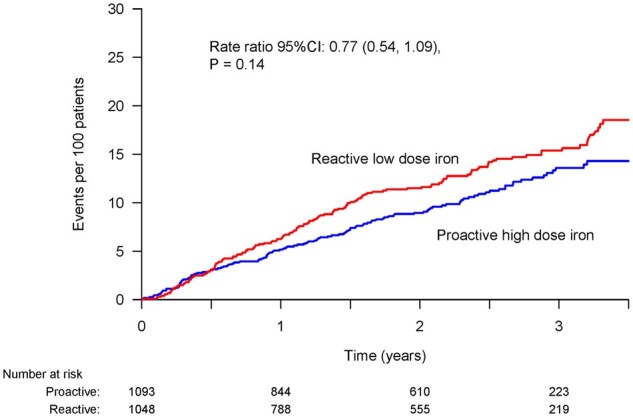
Effect of high- vs. low-dose IV iron on fatal and non-fatal MI (recurrent event analysis, i.e. first and subsequent events).

In the time-to-first event analysis, fatal or non-fatal MIs occurred in 78 of 1093 patients (7.1%; 3.5 events per 100 person-years) in the high-dose iron group and in 102 of 1048 patients (9.7%; 4.9 events per 100 person-years) in the low-dose group [hazard ratio (HR) 0.69, 95% confidence interval (CI) (0.52–0.93); *P* = 0.01] (*Table 3* and *Figure 1*). Fatal or non-fatal type 1 MI occurred in 5.7% in the high-dose group and 7.6% in the low-dose group (HR 0.71; 95% CI 0.51–0.99, *P* = 0.04). No reduction in non-fatal type 2 MIs was seen. In the recurrent event analysis, only fatal and non-fatal type 1 NSTEMIs were reduced by high-dose IV iron (*Table 3* and *Figure 2*).

**Table 3:** Effect of high-dose vs. low-dose iron on MI

	High-dose iron (*N* = 1093), *n* (%)	Rate (per 100 patient-years)	Low-dose iron (*N* = 1048), *n* (%)	Rate (per 100 patient-years)	HR/RR^a^ (95% CI)	*P*-value
Fatal or non-fatal MI						
Time-to-first	78 (7.1)	3.5	102 (9.7)	4.9	0.69 (0.52–0.93)	0.01
Recurrent	118	5.3	141	6.8	0.77 (0.54–1.09)	0.14
Fatal and non-fatal type 1 MI						
Time-to-first	62 (5.7)	2.8	80 (7.6)	3.9	0.71 (0.51–0.99)	0.04
Recurrent	84	3.8	109	5.3	0.71 (0.48–1.03)	0.07
Fatal and non-fatal type 2 MI						
Time-to-first	28 (2.6)	1.3	29 (2.8)	1.4	0.89 (0.53–1.50)	0.67
Recurrent	35	1.6	30	1.5	1.08 (0.62–1.87)	0.8
Fatal and non-fatal STEMI						
Time-to-first	9 (0.8)	0.4	13 (1.2)	0.6	0.64 (0.28–1.51)	0.31
Recurrent	10	0.5	14	0.7	0.67 (0.27–1.62)	0.37
Fatal and non-fatal NSTEMI						
Time-to-first	64 (5.9)	2.9	77 (7.4)	3.7	0.71 (0.51–1.00)	0.05
Recurrent	94	4.3	106	5.1	0.82 (0.55–1.22)	0.32

a For the time-to-first event analysis, the hazard ratio is presented; for the recurrent event analysis, the rate ratio is presented. Recurrent events include first and subsequent events.

## 4. Discussion

In patients who had started haemodialysis less than 12 months prior to enrolment, MI was a common event, with 8% having a fatal or non-fatal MI over 2 years of follow-up. Type 1 MIs and NSTEMIs were much more frequent than type 2 MIs and STEMIs. MIs were more common in this randomized trial than other cardiovascular events (e.g. heart failure hospitalization or stroke).^6^ IV iron administered in a high-dose regimen reduced acute fatal and non-fatal MI compared with low-dose iron. High-dose IV iron is the first therapy to reduce MIs in patients undergoing maintenance haemodialysis. These results suggest that the use of high-dose IV iron may be warranted to reduce MI.

Comparing the rate of acute MI over the last two decades is problematic as more sensitive biomarkers of myocardial damage (troponins) have been introduced. In more recent studies, clinical events, which would not previously have met the criteria for MI, have been classified as such. For example, at first glance, an incidence of MI of 10% over 2 years over 20 years ago in a US Medicare cohort on dialysis appears very similar to that seen in PIVOTAL. It is, however, very likely that if troponins had been used in these earlier studies more clinical episodes would have been classified as MIs and substantially higher rates reported.^10^ Observational data using hospital discharge coding suggested that NSTEMI is more common than STEMI in patients receiving dialysis.^11^ Our data from a randomized clinical trial using formally adjudicated events supports this but also demonstrates that most acute MIs are type 1 rather than type 2.

Until the current trial, no treatment had been shown to reduce acute MI in patients receiving haemodialysis. Notably, statin or other lipid-lowering therapies have not resulted in reductions in acute MI.^12^ In the PIVOTAL trial, patients who had an acute MI were more likely to have ESKD of diabetic or renovascular aetiology. It is possible that efforts to prevent MIs might be particularly targeted at groups with these aetiologies of renal disease.

In the PIVOTAL trial, the reduction in MI by high-dose compared to low-dose iron might be due to several factors. As this was a clinical trial our ability to determine mechanisms is limited. More IV iron is likely to result in more oxygen delivery; haemoglobin levels increased more rapidly in those receiving high-dose IV iron than those on the low-dose regimen.^6^ Such an action might be more likely to prevent type 2 than type 1 MI (by improving the ‘supply’ aspect of the oxygen ‘supply/demand mismatch’ in type 2 MIs), yet we saw more of an effect on type 1 MI. An effect secondary to more oxygen delivered by higher haemoglobin levels is supported by the greater HRs seen for time-to-first events than for recurrent events. The difference in haemoglobin is greatest between high- and low-dose arms early in the trial when the first events are happening and there is no difference in haemoglobin when recurrent events are taking place. If an increase in haemoglobin levels was the main mechanism of reduction in MI in PIVOTAL, it is difficult to explain why darbepoetin did not result in a reduction in MI in the TREAT trial.^13^ This trial was, however, conducted in a different population: patients with chronic kidney disease that were not receiving haemodialysis. It seems likely that the beneficial effects of high-dose iron are contributed to by other effects. An increase in platelets is known to be associated with iron deficiency.^14^ In the PIVOTAL trial, high-dose iron was associated with lower platelet levels than low-dose iron.^6^ This may be an additional or alternative mechanism explaining how IV iron reduces acute MIs. Acute MIs could also be reduced due to the direct effects of iron on the endothelium and circulating monocytes but data to support this hypothesis in patients on dialysis are lacking.

In the current trial, rates of death following non-fatal MI were very high (1- and 2-year mortality was 40% and 60%, respectively). Although these data are striking, these numbers appear to represent an improvement when compared with data from the 1980s and 1990s when extremely high mortality rates were reported (1-year mortality post-MI of 60%).^15^ Data from the USA reported a 2-year mortality of 71% as recently as 2008. On the other hand, it is possible that the lower mortality rate seen in the contemporary PIVOTAL trial does not reflect a reduction in mortality rates but from the inclusion of clinical events that are now classified as acute MIs because of the introduction of troponin. These acute MIs are likely to have been smaller and associated with better outcomes. In the current trial, the mortality rate in STEMI was very high but this must be verified in studies with larger numbers. Such high death rates after MI highlight that this is an area of major unmet need in cardiovascular medicine.

Whether or not the rate of acute MIs can be reduced in patients on haemodialysis can be improved is finally attracting some attention. ISCHEMIA-CKD 30172098 was an NHLBI funded trial that randomized 777 patients with estimated glomerular filtration rate <30 or on haemodialysis (53%) to a routine invasive strategy (i.e. routine coronary angiography) or a conservative approach with angiography only for ‘failure’ of optimal medical therapy.^16^ No benefit of a routine invasive strategy on the primary endpoint of death or MI was seen.

Very few trials have investigated the role of medical therapy to reduce MI in patients on haemodialysis. Patients on haemodialysis receiving 10 mg rosuvastatin had similar outcomes to placebo over a mean follow-up of 3.8 years.^17^ Patients with diabetes on dialysis had no benefit from atorvastatin 20 mg compared to placebo.^18^ Patients receiving dialysis have been excluded from trials establishing the beneficial effects of angiotensin-converting enzyme (ACE) inhibitors, angiotensin II receptor blockers (ARBs), and beta-blockers, meaning that it remains unclear as to whether these agents are efficacious or harmful.^19^ Over half of the patients in the current trial were receiving statins but prescription rates of ACE inhibitors or ARBs were low.

Another potential therapeutic target in patients receiving haemodialysis is hypertension. In the current trial, the mean systolic blood pressure was 145 mmHg. Patients with type 2 MIs had higher systolic blood pressures than those with type 1 MIs. The optimum blood pressure target for patients on haemodialysis is unknown. Perhaps trials of blood pressure lowering could reduce MIs.

### 4.1 Limitations

The PIVOTAL trial was conducted in the UK so the findings may not be generalizable to other countries or regions. The varying international nature of renal disease and cardiovascular disease could plausibly result in different results. Troponins can be chronically elevated in patients on dialysis and clinical presentation of MIs can be atypical.^20,21^ To overcome this, the Clinical Events Committee combined cardiology as well as nephrology expertise. The PIVOTAL trial design did not require documentation of all troponin values during each possible presentation with an MI. Cardiac magnetic resonance imaging can be useful to identify MIs but late gadolinium cannot be used in patients on haemodialysis. Systematic coronary angiography (including intracoronary imaging) can help to differentiate type 1 from type 2 MIs but is not practical to mandate during a large clinical trial. The capture of unrecognized or ‘silent’ MI was not performed during this trial. Other limitations of this analysis include a shared problem with all studies including type 2 MIs. Before a type 2 MI can be adjudicated a rise or fall in troponin must be seen. To see such a change, at least two measurements must be performed. In other words, if only one troponin (or no troponin at all) is measured, a type 2 MI cannot be diagnosed. Type 2 MIs are therefore always, to some extent, investigator-dependent events.

## 5. Conclusion

MIs occurred in 8% of patients over 2 years of follow-up in patients on maintenance haemodialysis. Most of these MIs are type 1 MIs and NSTEMIs. Mortality remains high after non-fatal MI (1- and 2-year mortality of 40% and 60%, respectively). High-dose vs. low-dose IV iron reduces MI in patients in their first year of haemodialysis.

## Supplementary material

Supplementary material is available at *Cardiovascular Research* online.

## Authors’ contributions

M.C.P.—first draft of the manuscript, study design, and Clinical Events Committee. P.S.J.—critical revision of the manuscript, study design, and Clinical Events Committee. E.C.—critical revision of the manuscript, study design, and Clinical Events Committee. P.B.M.—critical revision of the manuscript, study design, and Clinical Events Committee. M.R.M.D.—critical revision of the manuscript, study design, and Clinical Events Committee. M.R.—statistical analysis, critical revision of the manuscript, study design, and member of Clinical Events Committee. S.D.A.—Steering Committee, trial design, and critical review of the manuscript. S.B.—Steering Committee, trial design, and critical review of the manuscript. K.F.—Steering Committee, trial design, and critical review of the manuscript. P.A.K.—Steering Committee, trial design, and critical review of the manuscript. D.C.W.—Steering Committee, trial design, and critical review of the manuscript. C.R.V.T.—Steering Committee, trial design, and critical review of the manuscript. I.F.—Steering Committee, trial design, and critical review of the manuscript. J.J.V.Mc.M.—Steering Committee, trial design, and critical review of the manuscript. I.C.M.—Chief Investigator, Steering Committee, trial design, and critical review of the manuscript.

## Supplementary Material

cvab317_Supplementary_Data

## Data Availability

Although the PIVOTAL Steering Committee is not making the data available at present, the committee will consider public release of data in the future. Translational perspective Patients who have recently started haemodialysis are at high risk of myocardial infarction (MI). High-dose intravenous iron, when compared with low-dose intravenous iron, reduces MI by approximately one-third.
