# Supplementary material for: High-dose intravenous iron reduces myocardial infarction in patients on haemodialysis
Source: Cardiovasc Res. 2021 Dec 7;119(1):213–20. doi: 10.1093/cvr/cvab317 (PMC10022850; doi:10.1093/cvr/cvab317)
Supplement: cvab317_Supplementary_Data [file cvab317_supplementary_data.pdf]

Supplementary Appendix for "High-dose intravenous iron therapy reduces myocardial infarction in patients on hemodialysis: a secondary analysis from the PIVOTAL trial" MC Petrie et al

Page 2-6 - eFig S1 – Mortality after non-fatal MI (time 0 starts at the time of first MI for each patient)

Page 7 - eTable S1 Patients with a type 1 versus a type 2 MI: baseline characteristics

Page 8 - eTable S2 Patients with a STEMI versus a NSTEMI: baseline characteristics

Page 9 - eTable S3 – Causes of death

eFig S1 – Mortality after non-fatal MI (time 0 starts at the time of first MI for each patient)

Panel A – Mortality after all non-fatal MI

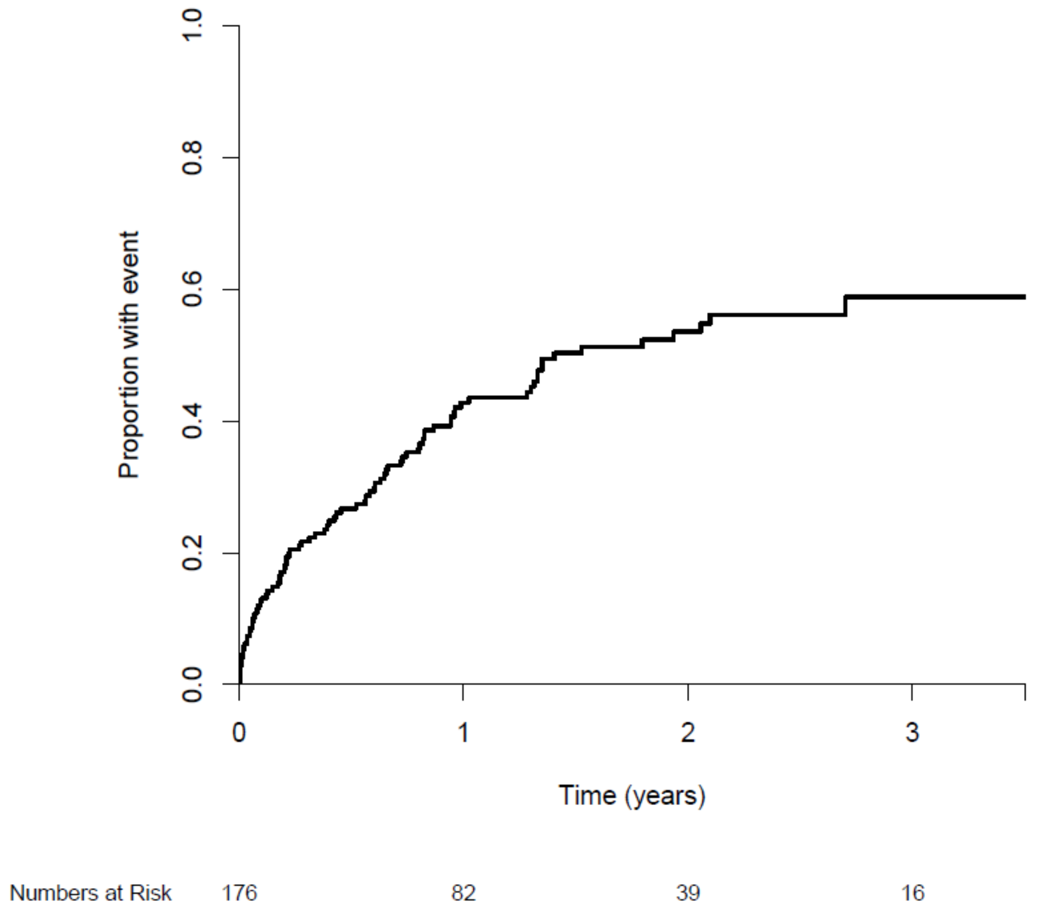

Panel B – Mortality after all non-fatal type 1 MI

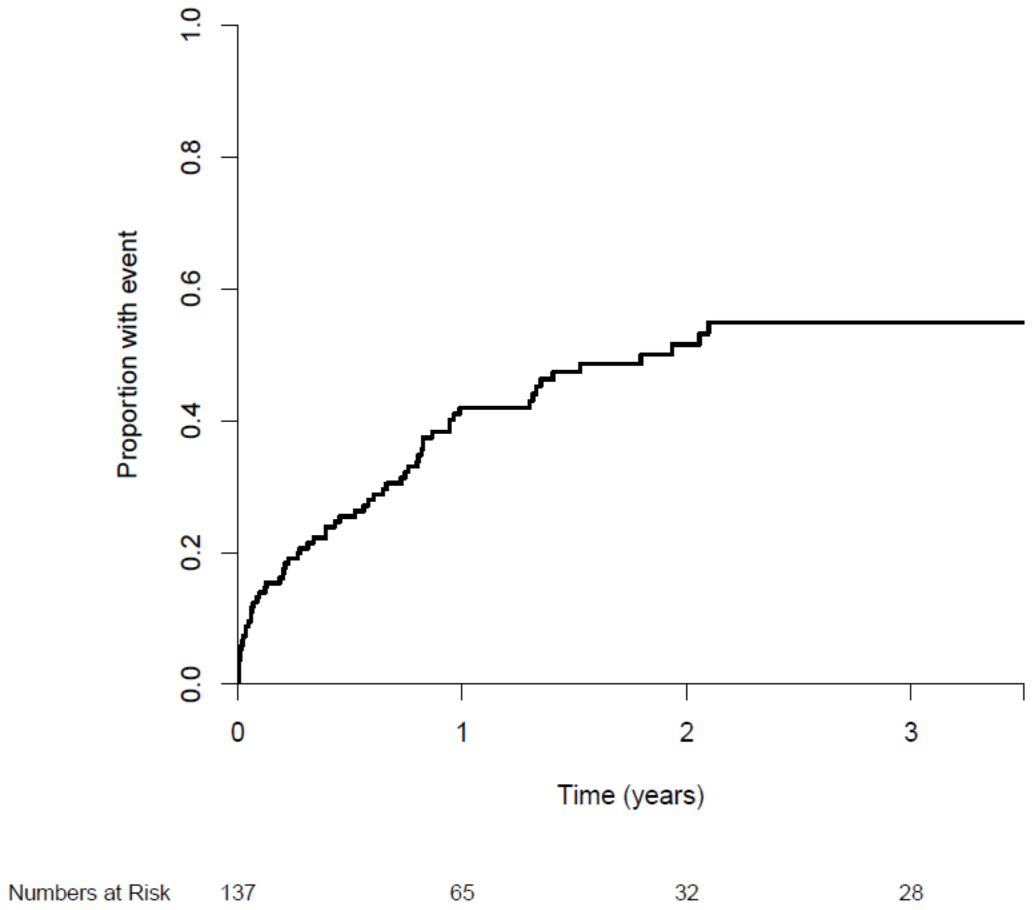

Panel C – Mortality after non-fatal type 2 MI

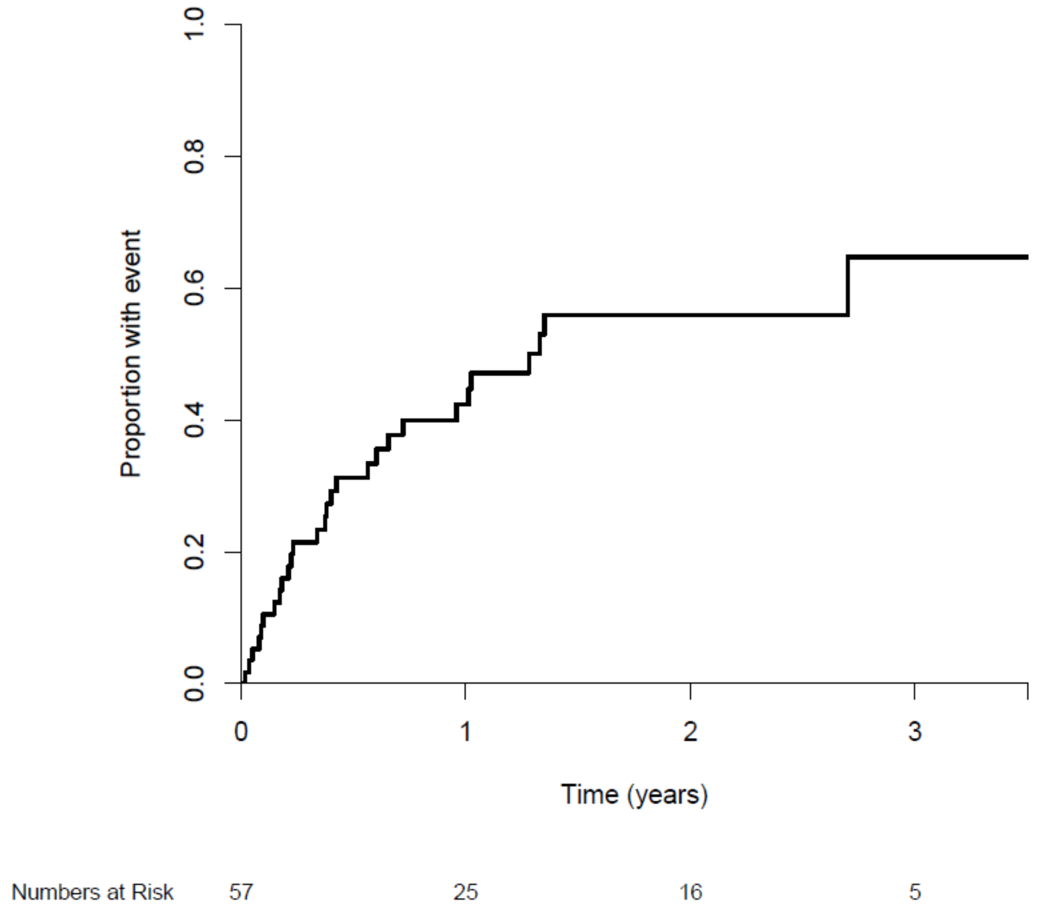

Panel D – Mortality after non-fatal NSTEMI

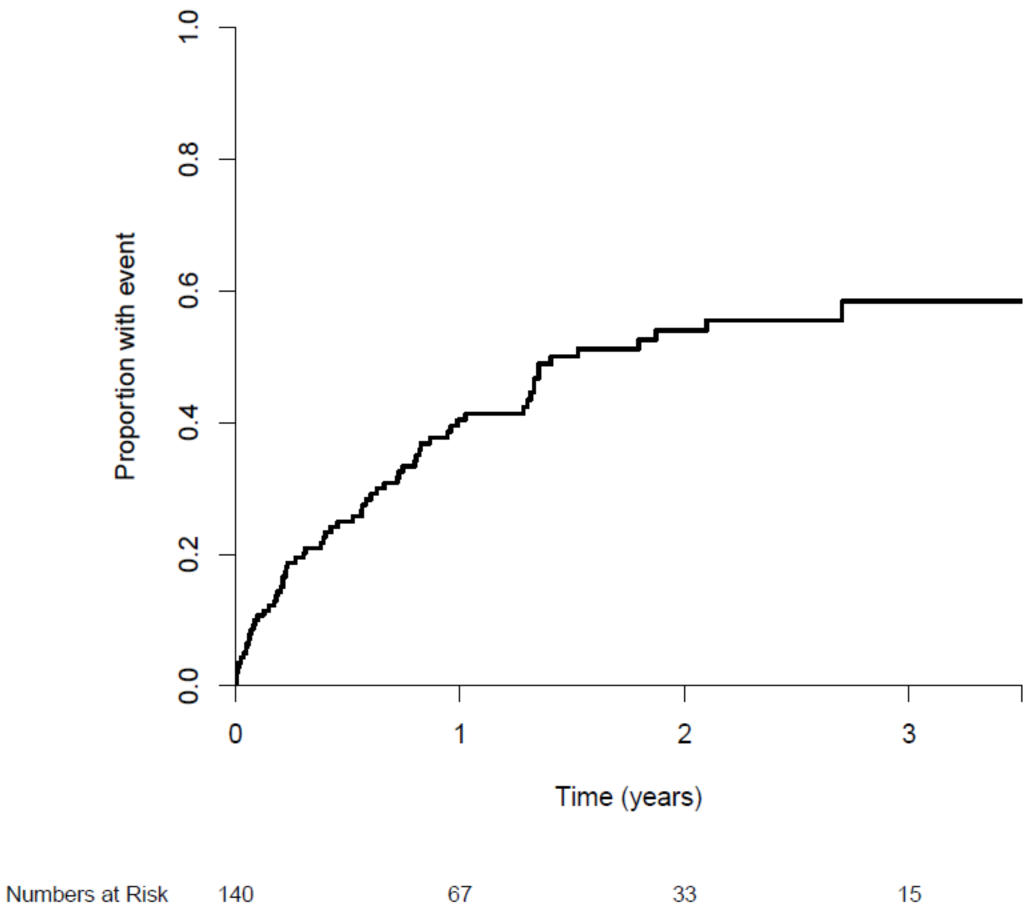

Panel E – Mortality after non-fatal STEMI

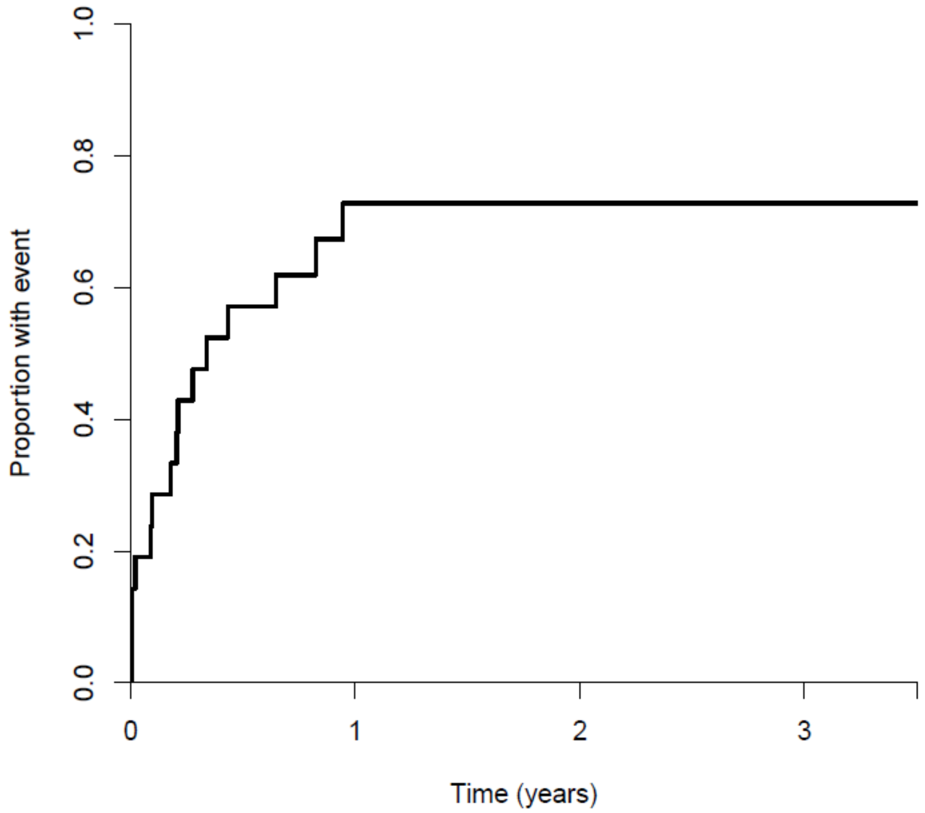

Numbers at Risk	21	6	6	6

eTable S1 Patients with a type 1 versus a type 2 MI: baseline characteristics

	**Type 1 MI**<br><br>**(N=133)**	**Type 2 MI**<br><br>**(N=44)**	**p**
Age, yr	67.4(11.8)	65.6(14.1)	0.39
Male sex, (%)	72.2	63.6	0.28
Race (%)			
White/European	77	89	0.53
Black/African descent	6	2	
Asian	14	9	
Other	2	0	
BMI, kg/m^2	28.7(5.8)	28.6(5.9)	0.87
Systolic BP, mm Hg	145(26)	156(21)	0.01
Median duration of dialysis, months	5.0(2.8,8.1)	4.8(2.5,7.9)	0.60
History, %			
Hypertension	81	80	0.87
AF	11	9	0.91
MI	17	30	0.04
PAD	19	7	0.08
HF	11	7	0.83
Stroke	13	11	1.00
Diabetes	65	64	0.90
Smoking status, %			
Never	57	50	0.33
Previous	27	39	
Current	16	11	
Laboratory measurements			
Haemoglobin	105(14)	106(13)	0.53
Ferritin	215(142, 293)	195(135, 294)	0.98
Transferrin saturation	19(15, 23)	20(15,25)	0.39
C-reactive protein	8(4,18)	7(4, 11)	0.20
CV medications, %			
β-Blocker	48	48	0.96
ACE inhibitor	13	11	0.80
ARB	10	5	0.36
Any diuretic	47	50	0.70
Statin	74	75	0.94
Any antiplatelet agent	65	75	0.24

For 3 MIs whether or not they were type 1 or type 2 MIs was unknown.

eTable S2 Patients with a STEMI versus a NSTEMI: baseline characteristics

	**STEMI**<br><br>**(N=15)**	**NSTEMI**<br><br>**(N=136)**	**p**
Age, yr	72.7(9.1)	66.7(12.5)	0.07
Male sex, (%)	73	68	0.78
Race (%)			
White/European	87	82	0.45
Black/African descent	0	4	
Asian	7	12	
Other	7	1	
BMI, kg/m^2	29.8(5.6)	28.5(5.8)	0.41
Systolic BP, mm Hg	144(20)	149(25)	0.53
Median duration of dialysis, months	6.2(2.6, 7.2)	5.0(2.8, 8.5)	0.90
History, %			
Hypertension	73	80	0.55
AF	13	10	0.004
MI	13	23	0.65
PAD	27	15	<0.001
HF	0	10	0.40
Stroke	13	15	0.23
Diabetes	60	63	0.81
Smoking status, %			
Never	60	51	0.21
Previous	40	32	
Current	0	18	
Laboratory measurements			
Haemoglobin	104(13)	105(13)	0.81
Ferritin	249(179,274)	207(130,300)	0.51
Transferrin saturation	19(14-27)	20(15-24)	0.77
C-reactive protein	7(5-12)	8(4-17)	0.95
CV medications, %			
β-Blocker	53	48	0.68
ACE inhibitor	13	12	0.69
ARB	7	9	1.00
Any diuretic	47	45	0.89
Statin	80	75	1.00
Any antiplatelet agent	67	69	1.00

For 29 events whether or not they were STEMI or NSTEMI was unknown.

eTable S3 – Causes of death

Causes of death	N	Rate/100 patient years
All-cause	515	12.1
Cardiovascular death	187	4.4
Fatal MI*	25	0.6
Non-cardiovascular death	222	5.2
Undetermined death	106	2.5

*fatal MI is a subcategory of CV death
